# Supplementary figures and images for: Factors impacting the pre-analytical quality of blood cultures—Analysis at a tertiary medical center
Source: PLoS One. 2023 Mar 16;18(3):e0282918. doi: 10.1371/journal.pone.0282918 (PMC10019732; doi:10.1371/journal.pone.0282918)

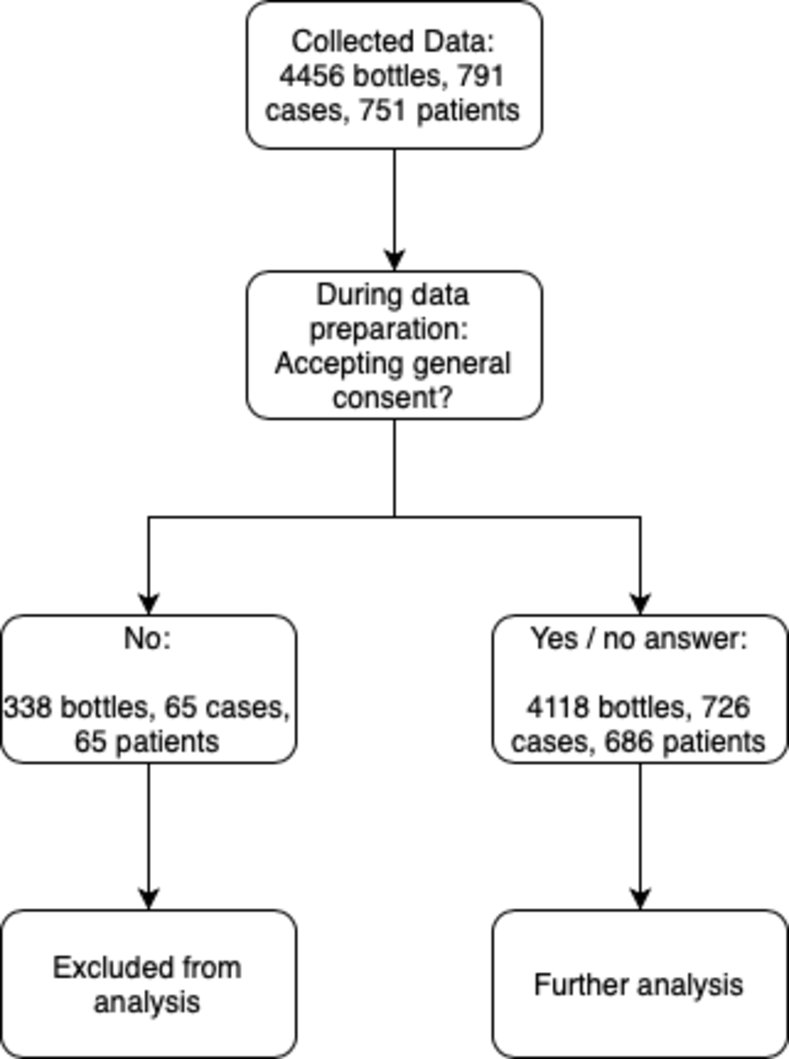

Supplement: S1 Fig — (TIF) [file pone.0282918.s001.tif]

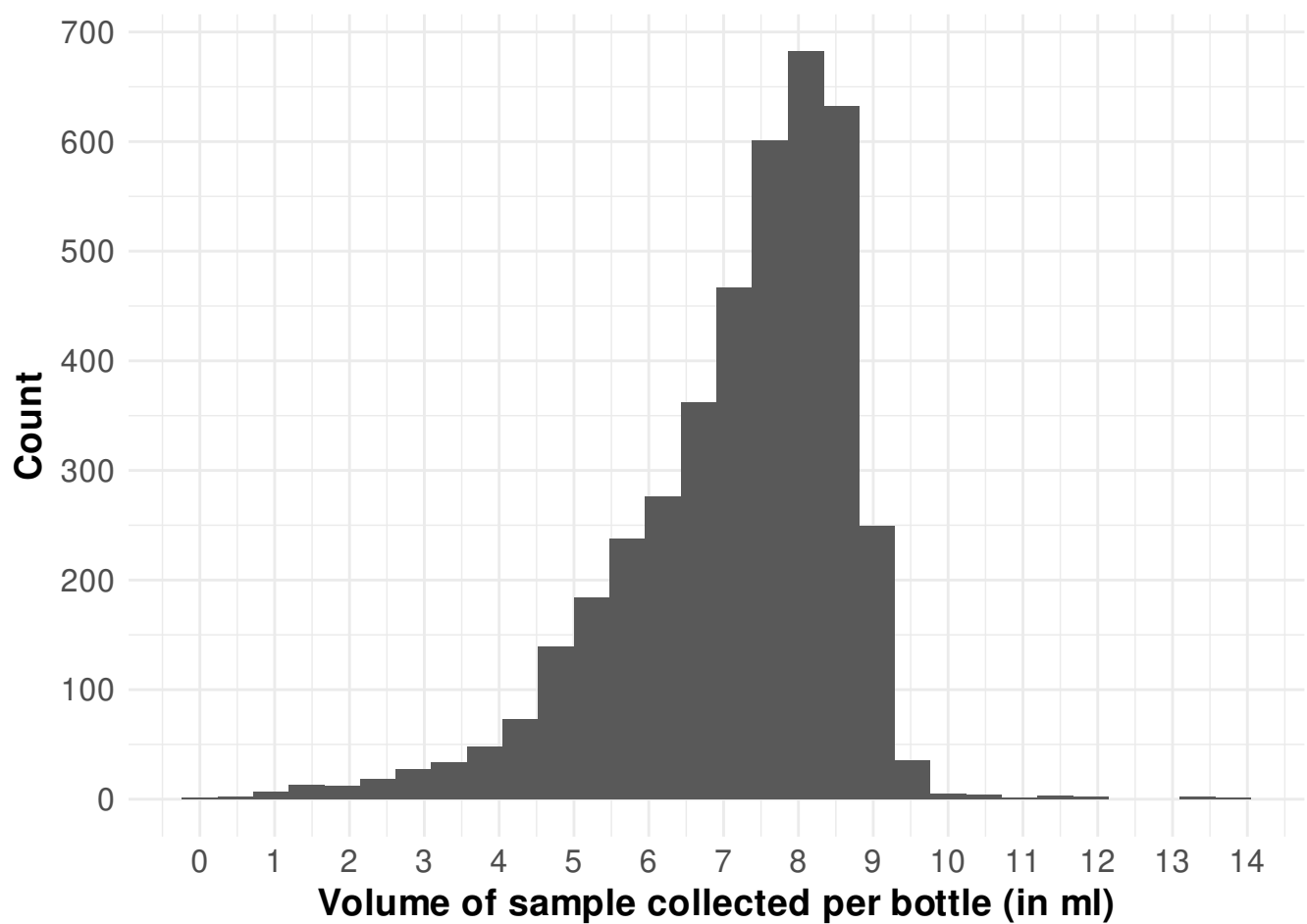

Supplement: S2 Fig — (PDF) [file pone.0282918.s002.pdf]

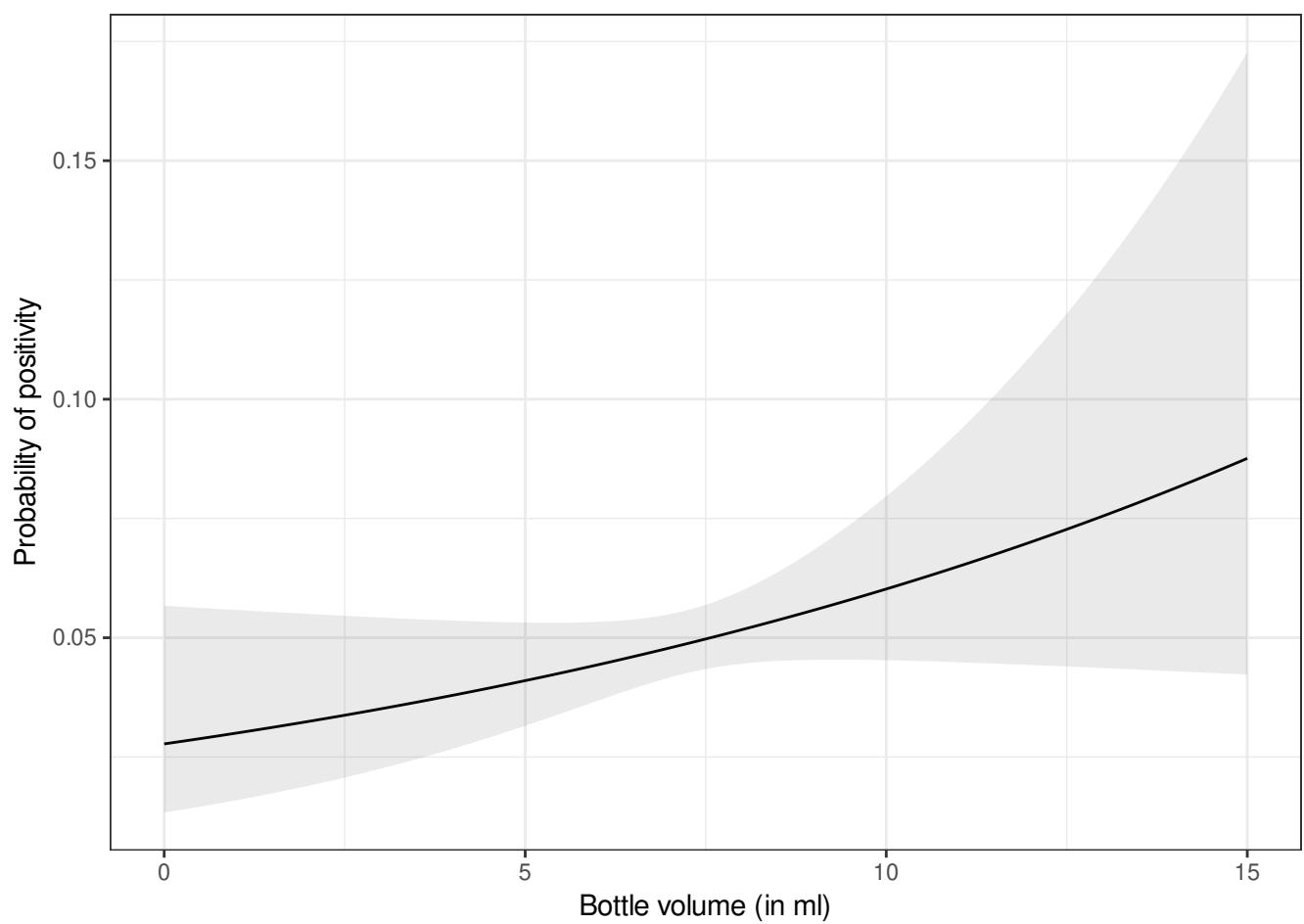

Supplement: S3 Fig — (PDF) [file pone.0282918.s003.pdf]
