# Supplementary material for: Factors impacting the pre-analytical quality of blood cultures—Analysis at a tertiary medical center
Source: PLoS One. 2023 Mar 16;18(3):e0282918. doi: 10.1371/journal.pone.0282918 (PMC10019732; doi:10.1371/journal.pone.0282918)
Supplement: S4 Fig — (PDF) [file pone.0282918.s004.pdf]

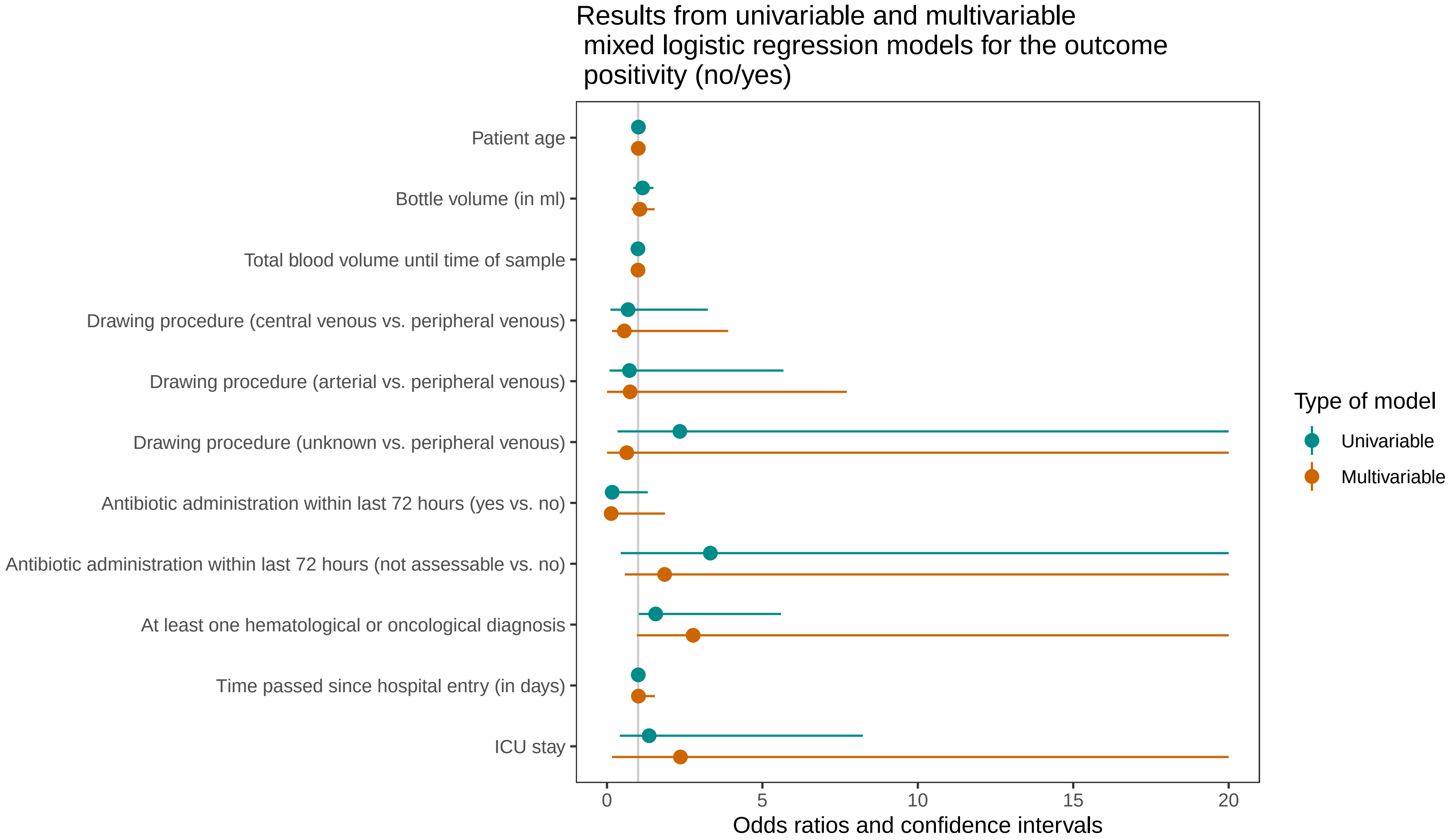

*Antibiotic administration within last 72 hours (not assessable vs. no) was capped at 20 to make it scalable with the other variables
