# Supplementary material for: Factors impacting the pre-analytical quality of blood cultures—Analysis at a tertiary medical center
Source: PLoS One. 2023 Mar 16;18(3):e0282918. doi: 10.1371/journal.pone.0282918 (PMC10019732; doi:10.1371/journal.pone.0282918)
Supplement: S5 Fig — (PDF) [file pone.0282918.s005.pdf]

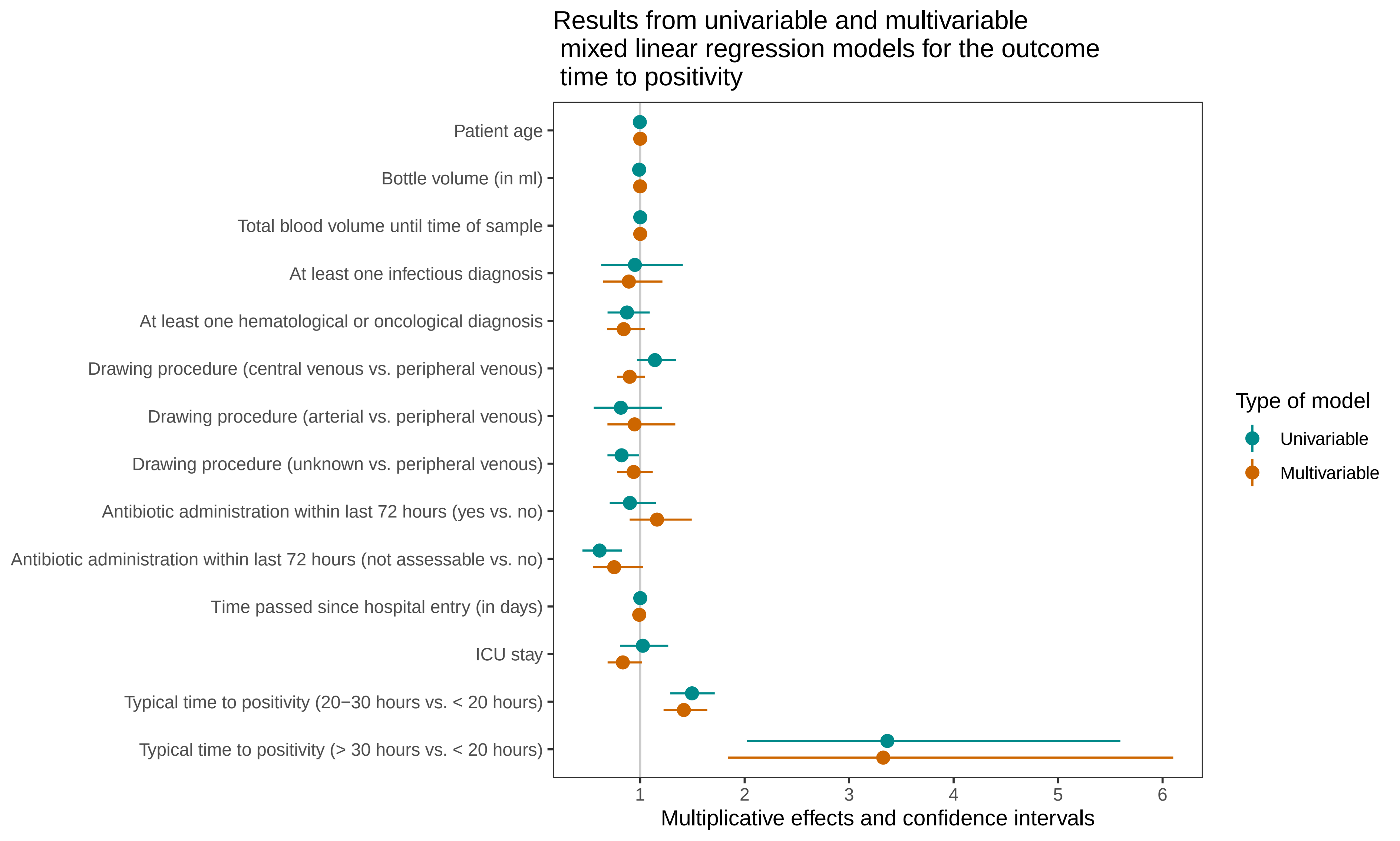
Results from univariable and multivariable
mixed linear regression models for the outcome
time to positivity
Patient age
Bottle volume (in ml)
Total blood volume until time of sample
At least one infectious diagnosis
At least one hematological or oncological diagnosis
Drawing procedure (central venous vs. peripheral venous)
Drawing procedure (arterial vs. peripheral venous)
Drawing procedure (unknown vs. peripheral venous)
Antibiotic administration within last 72 hours (yes vs. no)
Antibiotic administration within last 72 hours (not assessable vs. no)
Time passed since hospital entry (in days)
ICU stay
Typical time to positivity (20−30 hours vs. < 20 hours)
Typical time to positivity (> 30 hours vs. < 20 hours)
1
2
3
4
5
6
Multiplicative effects and confidence intervals
Type of model
Univariable
Multivariable
